# Urinary Tract Infections in Adults

**DOI:** 10.1100/tsw.2004.50

**Published:** 2004-06-07

**Authors:** Evan B. Cohn, Anthony J. Schaeffer

**Affiliations:** Department of Urology, Northwestern Medical School, Chicago, IL, USA

## Abstract

Urinary tract infection (UTI) is an exceedingly common problem prompting seven million office visits and one million hospitalizations in the United States each year (1). Advances in the understanding of both host and bacterial factors involved in UTI have led to many improvements in therapy. While there have also been advances in the realm of antimicrobials, there have been numerous problems with multiple drug resistant organisms. Providing economical care while minimizing drug resistance requires appropriate diagnosis, evaluation, and treatment of urinary tract infections.

## EVALUATION

Effective management begins by obtaining a careful history with attention given to current symptomatology, prior episodes of documented UTI, and risk factors that can initiate (e.g. urethral catheterization or sexual intercourse) or complicate (diabetes mellitus or pregnancy) a UTI. Urine should be carefully collected in order to minimize contamination by nonpathogens and sent for microscopic analysis and culture. A midstream voided specimen is generally adequate, but urethral catheterization or suprapubic aspiration may be necessary in an individual who cannot produce a clean specimen. Since UTI is a host inflammatory response to bacterial invasion, urinalysis will reveal pyuria (the presence of white blood cells in the urine) and bacteriuria. If there is bacteriuria with no pyuria in an asymptomatic patient, an infection is not present and therapy is usually not necessary. Urine is normally sterile and traditionally $10^5$ colony forming units per milliliter (cfu/ml) has been considered to be diagnostic of UTI. Frequent voids and alterations in patient hydration state will reduce bacterial counts to less than 105 in 30% of patients with UTI. Therefore urine culture which shows growth of 1000 or more cfu/ml should be investigated in symptomatic patients. Culture and antimicrobial susceptibility testing is not required for uncomplicated infection, but is essential to guide drug selection for complicated UTIs.

## PATHOGENESIS

Pathogenesis of urinary tract infection involves complex interactions between an organism, the environment, and the potential host. The majority of infections can be attributed to facultative anaerobes, the most common of which is Escherichia coli, which is responsible for 85% of infections in ambulatory patients and 50% of nosocomial infections. Proteus mirabilis, Klebsiella pneumonia, and Enterococcus fecalis, are the next most frequent isolates.

Most infections are caused by retrograde ascent of bacteria from the fecal flora via the urethra to the bladder and kidney. Hematogenous infection of the kidney by Gram positive organisms, such as Staphylococci, is possible though uncommon in normal individuals. There are urinary pathogen virulence factors that promote adherence to mucosal surfaces and subsequent infection (2). As shown in Figure 1 the bacteria usually express fimbriae or pili which mediate adherence to the epithelial cell receptors (3).

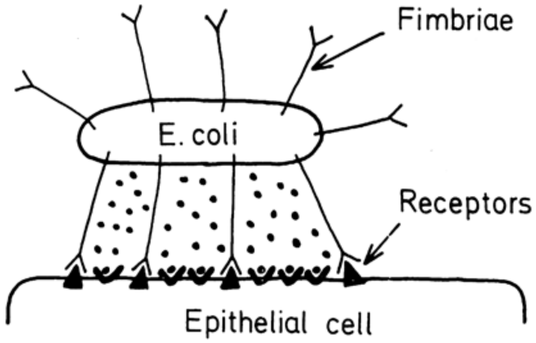

**FIGURE 1.** Bacterial Adhesins on pili (fimbrae) mediate attachment to specific epithelial cell receptors. (Courtesy of Cambell's Urology 7th edition, with the permission of W.B. Saunders)

Host factors such as the epithelial cell receptivity are also important in the infection process. For example, E. coli bound to vaginal epithelial cells from healthy controls less avidly than to vaginal epithelial cells from women with recurrent UTIs (4). This binding specificity correlated with binding to buccal mucosal cells, suggesting a genetic predisposition to urinary tract infection. Vaginal cell receptivity also varies as a function of hormonal status. Bacterial adherence was shown to be higher earlier in the menstrual cycle and in post menopausal women as compared to premenopausal women or postmenopausal women who are on estrogen replacement therapy (5, 6).

## CLASSIFICATION

Uncomplicated UTIs occur in patients with urinary tracts that are normal from both a structural and functional perspective. Most women in the outpatient setting present uncomplicated UTIs and respond promptly to short term, inexpensive oral antimicrobial therapy. Complicated UTIs occur in patients with any anatomic structural or functional abnormality that compromises therapy. Fever and chills are common along with other systemic symptoms and broad-spectrum therapy is required. Significant morbidity may occur if the abnormality is not identified and corrected. Infections are further characterized into the following categories: isolated infection, unresolved infection, and recurrent infection which is further divided into reinfection or bacterial persistence. Isolated infections are either initial episodes of infection or are separated by six months from other episodes of infections. Between 25-40% of women between 30-40 years of age and most men present with isolated infection. Unresolved infections occur in patients with whom therapy has been unsuccessful. Organisms resistant to the original antimicrobial will be isolated from a culture obtained during therapy. It has been shown that the organism was present in initial cultures but in amounts that escape usual detection methods. Causes of unresolved bacteriuria are listed in Table 1.

**TABLE 1**
**Causes of Unresolved Bacteriuria in Descending Order of Importance**

- Bacterial resistance to the drug selected for treatment
- Development of resistance from initially susceptible bacteria
- Bacteriuria caused by two different bacterial species with mutually exclusive susceptibilities
- Rapid reinfection with a new, resistant species during initial therapy for the original susceptible organism
- Azotemia
- Papillary necrosis from analgesic abuse
- Giant staghorn calculi in which the ìcritical massî of susceptible bacteria is too great for antimicrobial inhibition
- Self-inflicted infections or deception in taking antimicrobial drugs (a variant of Munchausenís syndrome)

Reinfections represent discrete episodes of infection in a patient who has received appropriate therapy and then has another infection initiated from outside the urinary tract. These represent 95% of recurrent urinary tract infections in women. Bacterial persistence occurs when therapy is subverted by sequestration of bacteria in a site that is protected from the drug. Recognizing bacterial persistence is important because the causes must be removed to achieve sterilization (Table 2).

**TABLE 2**
**Correctable Urologic Abnormalities That Cause Bacterial Persistence**

- Infection stones
- Chronic bacterial prostatitis
- Unilateral infected atrophic kidneys
- Ureteral duplication and ectopic ureters
- Foreign bodies
- Urethral diverticula and infected paraurethral glands
- Nonrefluxing, normal-appearing, infected ureteral stumps
- After nephrectomy
- Infected urachal cysts
- Infected communicating cysts of the renal calyces
- Papillary necrosis
- Paravesical abscess with fistula to bladder

## PRINCIPLES OF THERAPY

The goal of treatment of a urinary tract infection is sterilization of the urine, which should occur within hours of the first dose of an appropriately chosen antimicrobial (7). To accomplish this, selection of a suitable drug is critical. When choosing empiric therapy one must consider if the infection is complicated or uncomplicated, the spectrum of activity of the drug against the likely pathogen, potential untoward effects of the drug, patient compliance, and cost. Table 3 lists reasonable choices for empiric therapy. The duration of therapy should be guided by the presumed extent of tissue involvement and concentration of antimicrobial in the urine (8). It is desirable that the urine concentration of the drug exceeds the MIC of the infecting pathogen by the highest amount for the longest period of time.

**TABLE 3**
**Reliable Coverage of Antimicrobials Used in the Treatment of Urinary Tract Infections of Commonly Encountered Pathogens**

| Antimicrobial or antimicrobial class | Gram positive pathogens | Gram negative pathogens |
|---|---|---|
| Amoxicillin/ampicillin | streptococcus<br>enterococci | E. coli<br>P. mirabilis |
| Amoxicillin/clavulanate<br>Ampicillin/sulbactam | streptococcus<br>staphylococcus (not MRSA)<br>enterococci | E. coli, P. mirabilis,<br>H. influenzae, Klebsiella spp. |
| Antistaphyloccal penicillins | streptococcus<br>staphylococcus (not MRSA) | none |
| Antipseudomonal penicillins | streptococcus<br>enterococci | most including P.aeruginosa |
| 1st generation cephalosporins | streptococcus<br>staphylococcus (not MRSA) | E. coli, P. mirabilis, Klebsiella spp. |
| 2nd generation cephalosporins (cefamandole, cefuroxime, cefaclor) | streptococcus<br>staphylococcus (not MRSA) | E. coli, P. mirabilis,<br>H. influenzae, Klebsiella spp. |
| 2nd generation cephalosporins (cefoxitin, cefotetan) | streptococcus | E. coli, Proteus spp. (incl. indole+),<br>H. influenzae, Klebsiella spp. |
| 3rd generation cephalosporins | streptococcus | most ceftazidime, cefoperazone - P. aeruginosa |
| Aztreonam | none | most including P. aeruginosa |
| Aminoglycosides | staphylococcus (urine) | most including P. aeruginosa |
| Fluoroquinolones | none | most including P. aeruginosa |
| Nitrofurantoin | staphylococcus (not MRSA)<br>enterococci | many Enterobacteriaceae (not Providencia, Serratia, Acinetobacter), Klebsiella spp. |
| Trimethoprim/sulfamethoxazole | streptococcus<br>staphylococcus | most Enterobacteriaceae<br>(not P. aeruginosa) |
| Vancomycin | all including MRSA | none |

Understanding the three main mechanisms by which uropathogenic organisms manifest resistance to antimicrobials; natural resistance, selection of resistant mutants, and transferable resistance, can help guide therapy. Natural chromosomal resistance is exemplified by Proteus, which is never sensitive to nitrofurantoin. Selection of resistant mutants represents survival of a resistant strain that was present prior to therapy but survives due to underdosing. In practice, selection of resistant clones occurs in up to 10% of patients and can be mitigated by ensuring the appropriate dosing of the antimicrobial. Transferable resistance or R-factor resistance is caused by a plasmid-mediated, transferable element that confers multi-drug resistance. This manner of resistance is common and can be a particularly problematic issue with improper use of antimicrobials in the hospital setting (9). The transmission of R-factor occurs within the fecal reservoir of patients receiving beta-lactams, trimethoprim-sulfamethoxazole (TMP-SMX), aminoglycosides, and tetracyclines. Nitrofurantoin and the fluoroquinolones do not demonstrate R-factor mediated resistance.

## ANTIMICROBIALS

Trimethoprim-sulfamethoxazole, a combination that synergistically interferes with folate metabolism, is frequently used in the treatment of uncomplicated urinary tract infections. It is available in both parenteral and oral preparations and, with the notable exceptions of Pseudomonas and Enterococcus species, is effective against a broad range of urinary pathogens. Concentration of this economical drug in the urinary tract is excellent and the effect on the fecal flora is minimal. Skin rash and gastrointestinal complaints are the main side effects. Use of this drug in pregnancy and neonates under one month of age is contraindicated.

Nitrofurantoin disrupts carbohydrate metabolism and inhibits bacterial cell wall synthesis. It is effective against most uropathogens except Pseudomonas and Proteus (10). Since nitrofurantoin reaches high levels in the urine, but does not concentrate in tissue, it is ineffective in the treatment of infection involving solid organs such as pyelonephritis or prostatitis. There is limited interaction with the fecal reservoir resulting in minimal problems with resistance. It should not be used in patients with poor renal function because they have insufficient concentrating ability to deliver adequate levels to the urinary tract or as initial therapy in patients with complicated UTIs. In addition, hemolysis can be seen in patients that have glucose-6-phosphate dehydrogenase deficiency. Pulmonary fibrosis is one extremely rare but serious complication.

Aminopenicillins (i.e. ampicillin and amoxicillin) are frequently used in the treatment of a wide range of infectious processes including those in the urinary tract. This frequent use has resulted in up to 30% resistance that can be seen in clinical isolates (11). Extended spectrum synthetic penicillins and those compounded with beta-lactamase inhibitors are occasionally used in parenteral therapy for complicated pyelonephritis. Aztreonam, a monobactam, has a spectrum of activity similar to the aminoglycosides and is used primarily in patients with penicillin allergies.

Aminoglycosides, which inhibit bacterial RNA synthesis, are a useful class of drugs and, when combined with TMP-SMX or ampicillin, are part of first line therapy against pyelonephritis. They have largely maintained their spectrum of activity and, with appropriate monitoring of levels, the danger of renal toxicity can be minimized. Newer techniques of extended dose therapy which employ single daily doses of up to 7 mg/kg are equally efficacious as standard dosing and have a lower risk of nephrotoxicity (12).

Fluoroquinolones are inhibitors of DNA gyrase with a broad spectrum of activity that is ideal for empiric treatment of urinary tract infection (13, 14) . They are not, however, significantly more effective than less expensive antimicrobials in therapy for uncomplicated UTI. Coverage against Enterobacteriaceae and Pseudomonas species is excellent, with high levels of activity against Staphylococcal species, though Streptococcal coverage is marginal. Fluoroquinolones do have advantages in the treatment of complicated UTIs secondary to host factors, resistant organisms, or difficult-to-treat pathogens such as Pseudomonas aeruginosa (15). The relative resistance of anaerobes makes the impact on the vaginal flora and fecal reservoir minimal and initial reports of resistance were rare. Inappropriate use has caused an increased incidence of resistant strains (16). Fluoroquinolones are contraindicated in children because of theoretical danger in cartilage formation, but side effects are rare in adults. Although as a class fluoroquinolones are not nephrotoxic, their dosing must be adjusted in patients with renal failure.

## MANAGEMENT

### Upper Tract Infection

Acute pyelonephritis or inflammation of the kidney parenchyma and renal pelvis is a clinical diagnosis made on the basis of the classic symptoms of fever, chills, and flank pain. Other non-specific symptoms such as abdominal pain, nausea, vomiting, or diarrhea can accompany acute pyelonephritis. Therefore, a high index of suspicion must be maintained to make prompt diagnosis. Pyuria and bacteriuria and present and urine cultures are positive, although up to 20% of patients will have bacterial counts that are insufficiently high to register as positive on Gram's stain. Leukocytosis is common and less specific signs of inflammation, such as an elevated C-reactive protein or increased erythrocyte sedimentation rate, may also be present. Urine culture shows E. coli in 80% of cases, with Proteus, Enterobacter, Pseudomonas, Serratia, and Enterococcus also being common pathogens. Blood cultures are not indicated unless the patient is severely ill and/or compromised. Imaging studies, such as intravenous pyelogram, ultrasound, or CT scan are useful to rule out obstruction or focus of infection (e.g. abscess) that requires drainage. If the patient has a fever for greater than three days, is extremely ill, develops signs or symptoms of obstruction, or does not respond to initial antimicrobial therapy, imaging studies should also be obtained.

Patients that have symptoms consistent with pyelonephritis, but who are not septic or vomiting, may be treated on an outpatient basis with either trimethoprim-sulfamethoxazole or a fluoroquinolone for 14 days (Table 4). These patients must be closely monitored at home and re-evaluated if their clinical condition deteriorates. If clinical improvement is seen within 72 hours then a repeat urine culture should be performed 7-10 days after completion of therapy to document a cure. Lack of improvement at 72 hours or clinical deterioration at any point is indication to hospitalize and begin parenteral broad-spectrum antimicrobial therapy. Ampicillin and an aminoglycoside are a reasonable combination with acceptable coverage of E. coli and Pseudomas. However, if there is clinical suspicion of an ampicillin resistant organism, present in up to 30% of patients, trimethoprim-sulfamethoxazole (TMP-SMX) or vancomycin with an aminoglycoside provide more consistent coverage (17). Patients who present with sepsis or vomiting should be immediately hospitalized and receive parenteral broad-spectrum antimicrobial therapy for 14 days. After the patient becomes afebrile, therapy can be tailored from a parenteral form to an appropriate oral form based on the antimicrobial sensitivity profile. If symptoms persist for more than seven days or patients do not demonstrate improvement within 72 hours after the initiation of appropriate therapy, the possibility of renal or perinephric abscess, obstruction, or unknown urinary tract abnormality must be ruled out. The preferred imaging modality is CT scan, although ultrasound is also useful (18).

Patients with pyelonephritis that associated with a structural or functional abnormality should receive a 21-day course of therapy. Urine cultures should be obtained at 5-7 and 7-10 days of therapy, then 4-6 weeks after completion of therapy to document eradication of infection. Relapse rates of 10-30% for patients with pyelonephritis have been reported. Most patients respond to a second 14 day course, although occasionally a full 6 week course of antimicrobial therapy is required (19).

Chronic pyelonephritis causes kidneys to be small, contracted, or atrophic and is associated with cortical scarring. This entity is rare in a structurally and functionally normal urinary tract and has been associated with recurrent UTIs in young girls with vesicoureteral reflux (20). Adults with chronic pyelonephritis tend to have an obstructive etiology. It is likely that it is a combination of the host immunologic response along with the virulence of the infecting organism that is responsible for the damage to the kidney parenchyma.

As a clinical entity, chronic pyelonephritis can be insidious and almost an incidental finding during the evaluation of hypertension, renal failure, or even vague constitutional symptoms. Leukocytosis or pyuria are variably present but non-specific. The best single test for making the diagnosis is an intravenous pyelogram. Characteristic findings include atrophy, or scarring of the involved kidney, and clubbing of the calyces, which is a relatively specific finding. When the disease is unilateral there may be compensatory hypertrophy of the contralateral kidney. In children, where reflux may play a role, a voiding cystourethrogram should be obtained. This test is less useful in adults. Treatment centers around

**TABLE 4**
**Treatment Regimens for Acute Uncomplicated Pyelonephritis in Women**

| Circumstances | Route | Drug | Dosage | Frequency/Dose | Duration (in days) |
|---|---|---|---|---|---|
| Outpatient - Moderately ill, no nausea or vomitting | oral | TMP-SMX, | 160-800 mg | every 12 hours | 10-14 |
| | | ciprofloxacin, | 200 mg | every 12 hours | |
| | | enoxacin, | 400 mg | every 12 hours | |
| | | lomefloxacin, | 400 mg | every day | |
| | | norfloxacin | 400 mg | every 12 hours | |
| | | -or- | | | |
| | | ofloxacin | 200-300 mg | every 12 hours | |
| Inpatient - severely ill, possible sepsis | parenteral | TMP-SMX | 160-180 mg | every 12 hours | 14 |
| | | ampicillin and | 1 gm | every 6 hours | |
| | | gentamicin, | 1 mg/kg of body weight | every 8 hours | |
| | | ciprofloxacin, | 200-400 mg | every 12 hours | |
| | | ofloxacin, | 200-400 mg | every 12 hours | |
| | | -or- | | | |
| | | ceftriaxone | 1-2 gm | every day | |
| | | Take until afebrile, then take oral TMP-SMX or Fluoroquinolone | | | |
| Pregant | parenteral | ceftriaxone, | as above | as above | 14 |
| | | ampicillin and | as above | as above | |
| | | gentamicin, | as above | as above | |
| | | aztreonam, | 1 gm | every 8-12 hours | |
| | | -or- | | | |
| | | TMP-SMX * | as above | as above | |
| | | Take until afebrile, then take: | | | |
| | oral | cephalexin | 500 mg | every 12 hours | |

Modified from Stamm & Hooton, 1993

* Second trimester only appropriate, culture-guided antimicrobial therapy and the prevention of recurrent infection. Long-term treatment is sometimes necessary as the affected renal unit may have both structural abnormalities and a decreased ability to deliver antimicrobial to the kidney at therapeutic doses. Any remediable anatomic or obstructive problems should also corrected. Good results can be expected in appropriately treated patients, as evident in a study where patients with radiological findings consistent with bilateral chronic pyelonephritis had 95% and 86% survival at 5 and 10 years respectively and 100% at 10 years if the findings were unilateral (21).

Emphysematous pyelonephritis is infection of the kidney and surrounding tissues with a gas-forming organism, usually E. coli. The patients are exclusively adult, typically diabetic, and have a female predominance. The mortality rate is over 40%. Other than the classic presenting symptoms of fever, flank pain, and vomiting, the diagnosis is based on demonstration of intraparenchymal gas by plain abdominal films, ultrasound, or CT scan. Ten percent of cases are bilateral and a renal scan should be performed to evaluate degree of functional impairment of the involved side and status of the contralateral kidney. Aggressive management includes appropriate antimicrobial coverage, adequate diabetic control, and relief of obstruction if present. Nephrectomy is considered standard treatment, but recently, percutaneous drainage has been successful in carefully selected patients.

Renal abscess is a collection of purulent material involving the parenchyma of the kidney. It is more common in patients with calculi, obstruction, diabetes, or bladder dysfunction (22). The clinical presentation is similar to other infections of the upper tract. Prior to the widespread use of antimicrobials, hematogenous seeding by Gram positive organisms was the usual etiology, although currently Gram negative organisms predominate. Experimental evidence suggests that most of these Gram negative organisms gain access to the kidney in an ascending fashion. Urinalysis reveals pyuria and bacteriuria unless the abscess does not communicate with the collecting system. Leukocytosis and positive blood cultures are common. CT scan is the imaging test of choice because it provides superlative resolution and is able to detect abscesses earlier in their course. Differentiation between other pathology, such as tumor, is also superior with CT scan. Although there are instances where medical therapy has been successful, open or percutaneous drainage is usually instituted (23).

Perinephric abscess is an infection outside the kidney parenchyma but within Gerota's fascia. If Gerota's is breached, the entity is known as a paranephric abscess. The infection can follow antegrade or hematogenous seeding with Gram positive organisms from a distant antecedent infectious process. Gram negative organisms usually begin via a retrograde infection through the kidney, or via extension of infections from the gastrointestinal tract or chest. The diagnosis has historically been difficult, secondary to vague symptomatology. In a classic study by Thorley and associates of 52 patients with perinephric abscesses, more than half had symptoms greater than 14 days prior to hospital admission (24). Duration of symptoms greater than 5 days or persistence of symptoms for more than 4 days while on appropriate therapy were shown to be key to differentiating perinephric abscess from acute pyelonephritis. In this cohort, 25% of patients had a normal urinalysis and only one third had urine cultures that accurately identified the infecting organism. It is clear that delay in diagnosis markedly increased mortality. The standard treatment is parenteral antimicrobial therapy and surgical drainage or nephrectomy. With improved imaging techniques, percutaneous drainage can be effective in selected patients.

Xanthogranulomatous pyelonephritis, XGP, is exceedingly rare. The name originates from the xanthoma cells, which are lipid-laden macrophages that participate in the inflammatory reaction. XGP is characterized by renal calculi, infection (usually E. coli or Proteus), and obstruction. The average age of onset is between 50-70 years and women are infected three times more often than men. XGP can be a difficult diagnosis to make preoperatively as it can appear similar to a renal mass on CT scan. Nephrectomy should be performed and all involved tissue removed to allow adequate healing.

## Lower Tract Infection

Cystitis, an infection of the urinary bladder, affects between 4-6 million women each year and it is estimated that over 25% of women in the 20-40 age group have had a urinary tract infection. Most infections in women are uncomplicated, whereas in men complicated infections predominate. Presenting symptoms include dysuria, frequency, urgency, and suprapubic pain or tenderness. Patients may also occasionally complain of hematuria or foul-smelling urine. The history should identify those with other processes causing similar symptoms such as vaginosis, urethral syndromes, sexually transmitted diseases, or other non-inflammatory processes. A presumptive diagnosis of acute bacterial cystitis can be made based on a thorough history and after urinalysis reveals pyuria and bacteriuria. Indirect dip-stick test for nitrite or leukocyte esterase is less sensitive.

Urine culture is the gold standard for assessing infection but it need not always be ordered. Specifically, in women who have a history and urinalysis consistent with uncomplicated cystitis, effective empiric therapy can be instituted prior to having culture results available. Obtaining pre-treatment culture has been shown to increase cost by 40% while only decreasing overall symptom duration by 10% (25). Urine culture with sensitivities should be obtained: in patients where the diagnosis is not certain; if there is a history recent antimicrobial therapy; if the symptoms are recurrent or have lasted more than 7 days; and in pregnant, elderly, diabetic, or male patients. The threshold of $10^5$ cfu/ml, usually considered to be diagnostic of UTI, should be lowered to $10^2$ cfu/ml in patients who have symptoms consistent with UTI.

Choice of appropriate therapy is guided by whether the infection is complicated vs. uncomplicated, nosocomial vs. community acquired, as well as the sex of the patient. For uncomplicated acute bacterial cystitis in women, a three day course of TMP-SMX is adequate empiric treatment (26). Fluoroquinolones should be reserved for patients with recurrent infections, allergy or sensitivity to TMP-SMX, and those in whom organisms resistant to TMP-SMX are suspected. Women with uncomplicated UTI should have follow-up urinalysis 7-10 days post therapy and all others should have a urinalysis and culture to document cure. The rational for antimicrobial therapy in male patients is similar, although the duration of therapy should be 7 days. Table 5 shows a management strategy for acute cystitis and Table 6 and Table 7 review treatment regimens for acute cystitis and complicated lower tract infection.

**TABLE 5**
**Management Strategy for Acute Cystitis**

Recurrent infections of the lower tract are likely to originate from outside the urinary tract. Obtaining an accurate history of prior infecting organisms, hormonal status, relationship of infection to sexual intercourse, and diaphragm or spermacide are all important. Radiologic evaluation of the upper tracts is not indicated in otherwise healthy patients unless there is unexplained hematuria, obstructive symptoms, neurogenic bladder dysfunction, or diabetes. Cystoscopy is indicated in those with frequent infections and

**TABLE 6**
**Treatment Regimens for Acute Cystitis**

| Circumstances | Route | Drug | Dosage | Frequency/Dose | Duration (in days) |
|---|---|---|---|---|---|
| **Women** Healthy | oral | TMP-SMX, | 160-800 mg | every 12 hours | 3 |
| | | TMP, | 100 mg | every 12 hours | |
| | | microcrystalline nitrofurantoin, | 100 mg | four times a day | |
| | | ciprofloxacin, | 250 mg | every 12 hours | |
| | | enoxacin, | 400 mg | every 12 hours | |
| | | lomefloxacin, | 400 mg | every day | |
| | | -or- | | | |
| | | norfloxacin | 400 mg | every 12 hours | |
| Symptoms for >7 days, recent urinary tract infection, age >65 yrs, diabetes, diaphragm use | | TMP-SMX | as above | as above | 7 |
| | | -or- | | | |
| | | Fluoroquinolone | as above | as above | |
| **Pregnancy** | oral | amoxicillin, | 250 mg | every 8 hours | 7 |
| | | cephalexin, | 500 mg | four times a day | |
| | | microcrystalline nitrofurantoin, | as above | as above | |
| | | -or- | | | |
| | | TMP-SMX | as above | as above | |
| **Men** Healthy and less than 50 yrs old | oral | TMP-SMX,* | as above | as above | 7 |
| | | -or- | | | |
| | | Fluoroquinolone | as above | as above | |

Modified from Stamm & Hooton, 1993

* Second trimester only symptoms of obstruction, bladder dysfunction, or fistula. Patients who have rapidly recurrent infections with the same organism should be evaluated to identify a focus of bacterial persistence, such as a calculus or ureteropelvic junction obstruction, which must be removed or corrected to prevent further episodes. Patients with rapidly recurrent infections with the same organism should be evaluated to identify a focus of bacterial persistence, such as a calculus or ureteropelvic junction obstruction, which must be removed or corrected to prevent further episodes.

Prophylactic antimicrobials are used to prevent reinfection from outside the urinary tract. This is in distinction to suppressive therapy which is used to repress a focus of bacterial persistence. Suppressive therapy is reserved for patients who cannot be rendered free of the focus of persistence or are poor candidates for such treatment. The basis for prophylaxis is the eradication of pathogenic bacteria from anatomic reservoirs (vaginal introitus and feces) without promoting bacterial resistance. A single bedtime dose of nitrofurantoin (50 mg), 1/2 tablet of TMP-SMX, or cephalexin (250 mg) are all effective choices. Duration of therapy should be six to twelve months. If the patient experiences a symptomatic infection while on prophylaxis, full therapeutic dosing with the agent or another drug should be instituted, returning to the usual regimen after completion of the full course. If the patient has a symptomatic UTI after cessation of therapy, prophylaxis may be restarted. Following this regimen has been shown to reduce the reinfection rate from 2.0-3.0 per patient year to 0.1-0.4 per patient year (27). If infection has a temporal relationship to sexual intercourse, then a single post-coital dose of TMP-SMX, nitrofurantoin, or cephalexin have been shown to reduce the incidence of infection (28).

**TABLE 7**
**Treatment Regimens for Complicated Urinary Tract Infections**

| Circumstances | Route | Drug | Dosage | Frequency/Dose | Duration (in days) |
|---|---|---|---|---|---|
| Outpatient - Moderately ill, no nausea or vomitting | oral | ciprofloxacin, | 500 mg | every 12 hours | 10-14 |
| | | enoxacin, | 400 mg | every 12 hours | |
| | | lomefloxacin, | 400 mg | every day | |
| | | norfloxacin -or- | 400 mg | every 12 hours | |
| | | ofloxacin | 200-300 mg | every 12 hours | |
| Inpatient - severely ill, possible sepsis | parenteral | ampicillin and | 1 gm | every 6 hours | 14-21 |
| | | gentamicin, | 1 mg/kg of body weight | every 8 hours | |
| | | ciprofloxacin, | 200-400 mg | every 12 hours | |
| | | ofloxacin, | 200-400 mg | every 12 hours | |
| | | ceftriaxone, | 1-2 gm | every day | |
| | | ticarcillin-clavulanate, | 3.2 gm | every 8 hours | |
| | | imipenem-cilastatin, -or- | 250-500 mg | every 6-8 hours | |
| | | aztreonam | 1 gm | every 8-12 hours | |
| | | Take until afebrile, then take oral fluoroquinolone | | | |

Some patients are reluctant to take long term prophylaxis, particularly those with relatively infrequent infections. For these patients, intermittent self-start treatment may be an option. Patients identify their own episodes of infection by their symptoms. After a midstream urine culture is obtained on a special dip slide device, a three-day course of full-dose antimicrobial is started. The culture should be transported to the office within 72 hours. If the initial culture is positive, it is important to check a repeat culture 7-10 days after therapy to document eradication of infection. In a randomized crossover study, 35/38 symptomatic patients had urine that was consistent with infection microscopically and 30/35 infections improved clinically with a single dose of TMP-SMX (29). It is important to obtain intratherapy cultures in patients that do not respond appropriately to initial therapy and also evaluate patients that have persistent symptoms despite negative cultures.

Other approaches to prophylaxis are also being investigated. In postmenopausal women, topical estrogen replacement has been shown to restore the vaginal pH to its premenopausal range, promote recolonization with Lactobacilli, decrease colonization with E. coli, and subsequently result in fewer infections (30). Another approach currently in clinical trials involves the use of a "vaccine" composed of a vaginal suppository of non-uropathogenic strains of bacteria (31).

Prostatitis is actually a constellation of syndromes including acute bacterial prostatitis, chronic bacterial prostatitis, and abacterial prostatitis. Acute bacterial prostatitis is rare, constituting only 5% of all cases of prostatitis, and is usually caused by coliform bacteria. The infection causes perineal or low back pain, high fever, chills, dysuria, and other constitutional symptoms. Rectal exam reveals an extremely tender, inflamed, boggy prostate. Prostatic massage should not be performed as there is high risk for making the patient bacteremic and urine culture is usually positive for the causative organism. A fluoroquinolone, TMP-SMX, or ampicillin with an aminoglycoside are excellent first choice therapies, all obtaining therapeutic levels in the prostate. When afebrile, the patient may be converted to oral TMP-SMX or a fluoroquinolone and therapy should be continued for 30 days. Urinary retention, which commonly occurs, should be managed by placement of a suprapubic catheter to avoid urethral instrumentation and its attendant complications.

## CONCLUSION

Treatments for urinary tract infection have changed markedly over the last half century. Improvements in the antimicrobial armamentarium have been met by increased emergence of resistant bacterial strains. Encountering immunocompromised patients is increasingly common and there is ever increasing emphasis on providing therapy that is not only effective, but economical. Tailoring the principles reviewed here to individual patients can help the practicing urologist meet these challenges.
